# Primary Oral Tuberculosis as an Indicator of HIV Infection

**DOI:** 10.4061/2011/893295

**Published:** 2010-12-20

**Authors:** R. A. G. Khammissa, N. H. Wood, R. Meyerov, J. Lemmer, E. J. Raubenheimer, Liviu Feller

**Affiliations:** ^1^Department of Periodontology and Oral Medicine, School of Oral Health Sciences, Faculty of Health Sciences, University of Limpopo (Medunsa Campus), Medunsa 0204, South Africa; ^2^Department of Oral Pathology, School of Oral Health Sciences, Faculty of Health Sciences, University of Limpopo (Medunsa Campus), Medunsa 0204, South Africa

## Abstract

We present a case of primary oral tuberculosis that led to the diagnosis of HIV infection. Our patient had clinically nonspecific ulcers on the labial mucosa and on the ventral surface of the tongue which were diagnosed as being tuberculous only on histological examination. This raised the suspicion of HIV infection that was subsequently confirmed by blood tests. The oral lesions resolved after 4 weeks of antituberculosis treatment. Some aspects of the pathogenesis of HIV-tuberculosis coinfection are discussed.

## 1. Introduction

It is estimated that one-third of the world's population are infected with M*ycobacterium tuberculosis* (M.tb). Tuberculosis (TB) is a major cause of morbidity and mortality in developing countries [1]. Only about 11% of the world's population live in Africa, but they account for about 30% of all M.tb-infected subjects in the world [2].

HIV-seropositive subjects are at increased risk of acquiring M.tb infection, of re-activation of latent M.tb infection, and of more rapid progression of active TB than are HIV-seronegative subjects. Thus, the epidemic of HIV infection in Africa is undoubtedly a major factor accounting for the high prevalence of TB in Africa. In HIV-seropositive subjects co-infected with M.tb, the TB accelerates the course of HIV disease [3, 4]. 

In sub-Saharan Africa, about 80% of subjects with TB are coinfected with HIV, and it is estimated that in South Africa 30% of HIV-seropositive subjects have active TB [4]. M.tb re-activation may occur in the context of only moderate immunodeficiency, but the risk of TB is generally inversely related to the CD4+ T cell count and is greatest when the CD4+ T cell count dips below 200 cells/mm^3^ [3, 4].

Transmission of TB is by inhalation of airborne infectious droplets from persons with active pulmonary TB when they cough, sneeze, or speak. Extrapulmonary active TB is not infectious, unless it affects parts of the body such as the skin or the mouth from which M.tb can be transmitted by direct contact [5].

M.tb in the oral fluids of people with pulmonary TB is common, but oral TB is uncommon. This is probably owing to the protection of the intact oral epithelial barrier against M.tb penetration, and to the antibacterial properties and the flushing effects of the saliva [6].

Oral TB usually results from secondary inoculation of oral mucosa breached by any type of ulceration or by minor masticatory trauma, by infected sputum, or by haematogenous dissemination from other infected sites [7, 8]. 0.1%–0.5% of subjects with pulmonary TB will develop secondary oral TB affecting most commonly the tongue, followed by the palate, the lips, the buccal mucosa, and the gingiva [7–10]. It usually manifests as nonhealing ulcers but may also occur as nodules, granulomata or fissures, or as tuberculous osteomyelitis of the jaws [7, 8, 11]. Oral TB ulcers are usually single rather than multiple; the tuberculous ulcer has an indurated, irregular, undermined margin, and a necrotic base [12]. 

Rarely, oral TB may be brought about by primary infection by direct inoculation. The site most commonly affected is the gingiva where primary TB appears as a diffuse erythematous patch or as diffuse gingival enlargement [10, 11]. 

Subjects with HIV-M.tb co-infection more frequently have extrapulmonary TB than do HIV-seronegative subjects, lymph nodes and central-nervous system being the sites most commonly affected [1, 4]. Despite the increasing prevalence of extrapulmonary TB in HIV-seropositive subjects, the frequency of oral TB has not increased [1, 4].

We present a case of primary oral TB that led to the diagnosis of HIV infection.

## 2. Case Report of Primary TB of the Oral Mucosa

A 33-year-old black female attended the oral medicine clinic at the Medunsa Oral Health Centre, University of Limpopo, complaining of a painful sore on the upper left labial mucosa of about 3 weeks duration. She appeared to be in good health. The patient did not smoke or drink alcohol, and she claimed to be HIV-seronegative as she had been tested for HIV infection about three months previously.

**Figure 1:**
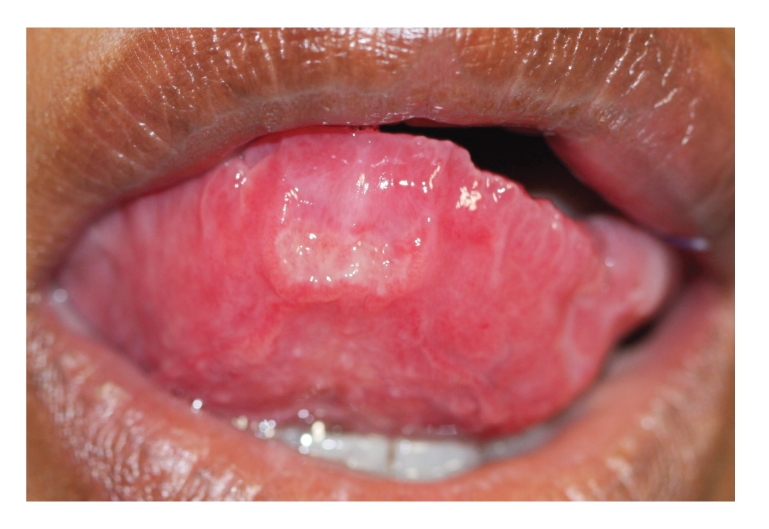
Ulcer on the ventrum of the tip of the tongue, with slightly elevated margins and a wide zone of surrounding erythema.

**Figure 2:**
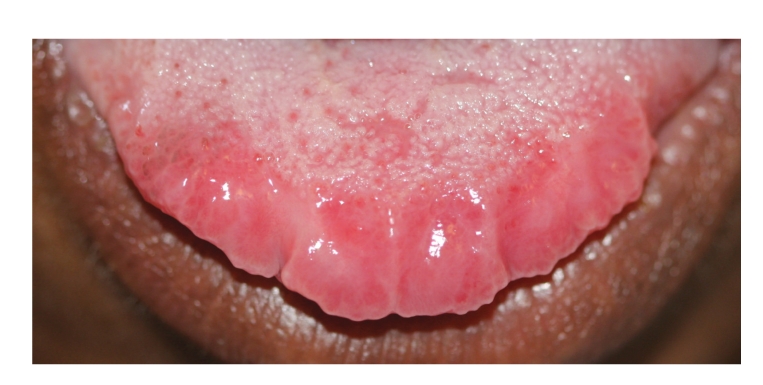
Dorsum of anterior one-third of the tongue with erythematous, lobulated appearance.

There were two ulcers surrounded by a wide area of erythema, one on the upper left labial mucosa that was painful, and one on the ventral surface of the tip of the tongue (Figure 1). The margins of both ulcers were slightly elevated and indurated. The dorsal surface of the anterior margin of the tongue was hyperaemic with a lobulated appearance (Figure 2).

**Figure 3:**
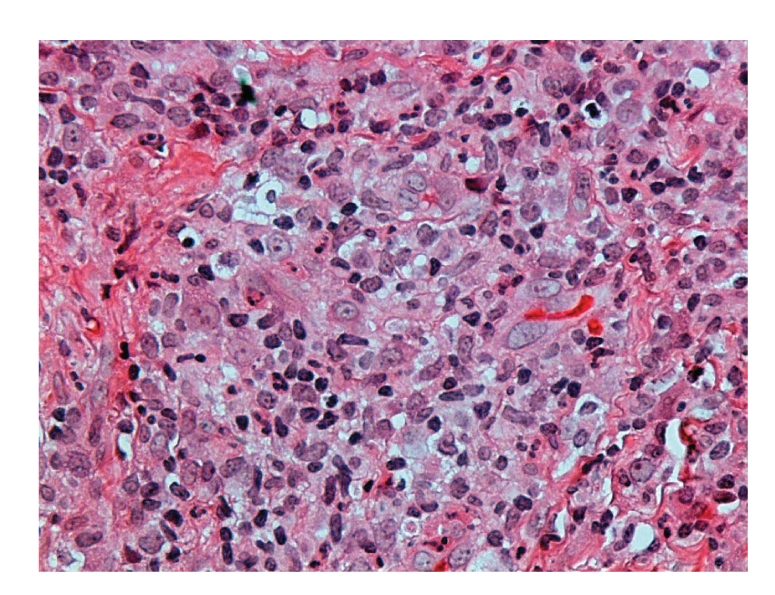
The chronic granulomatous lesion in the submucosa of the lip (H&E stain, ×300).

**Figure 4:**
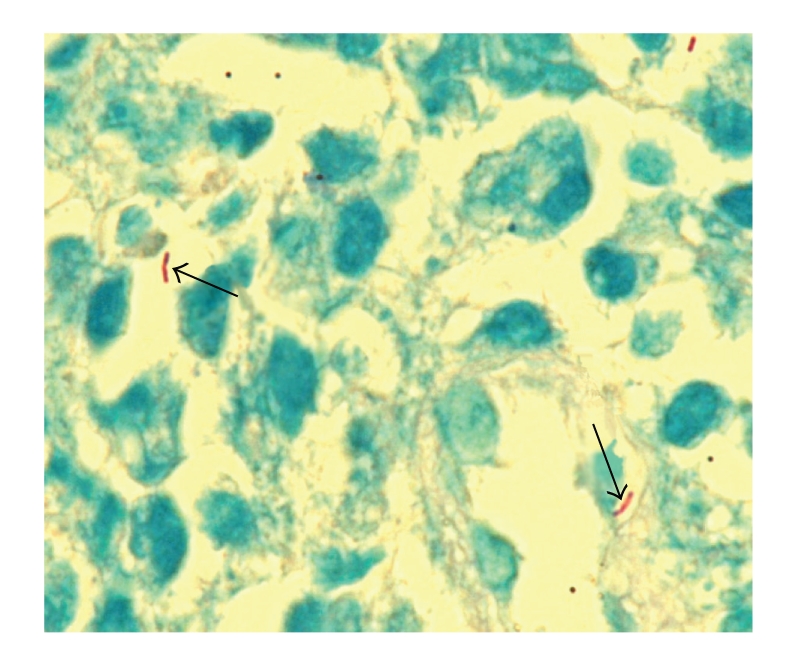
Ziehl-Neelsen stain showing two acid-fast bacilli (arrows, ×1000).

Histological examination of the labial mucosal ulcer showed necrotic tissue and chronically inflamed granulation tissue. Scattered epithelioid cells were present, and Ziehl-Neelsen staining revealed the presence of acid fast bacilli leading to the final diagnosis of oral tuberculosis (Figures 3 and 4).

The patient was referred to the Department of Internal Medicine at the Dr. George Mukhari Hospital. She proved to be HIV-seropositive with a CD4+ T cell count of 429 cells/mm^3^. The general physical examination was unremarkable, the chest radiograph did not show any evidence of tuberculosis, and other laboratory investigations that were deemed necessary were all within the range of normality. A nine-month anti-TB drug regimen of isoniazid, rifampicin, pyrazinamide, and ethambutol, was prescribed.

The patient did not receive antiretroviral treatment for a reason to be explained below.

**Figure 5:**
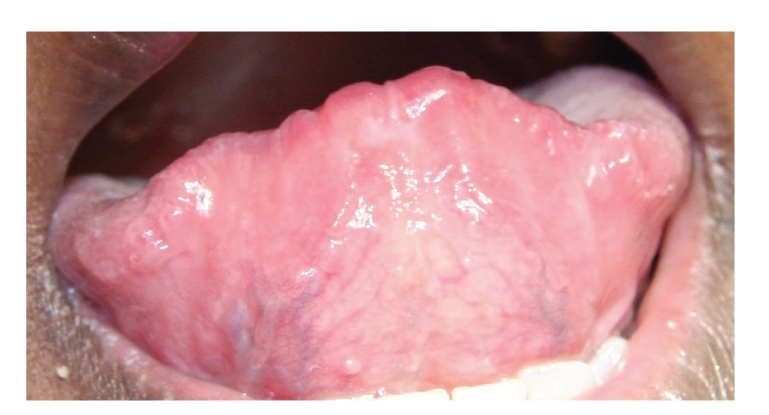
The same site as shown in Figure 1, four weeks after starting antitubercular treatment.

**Figure 6:**
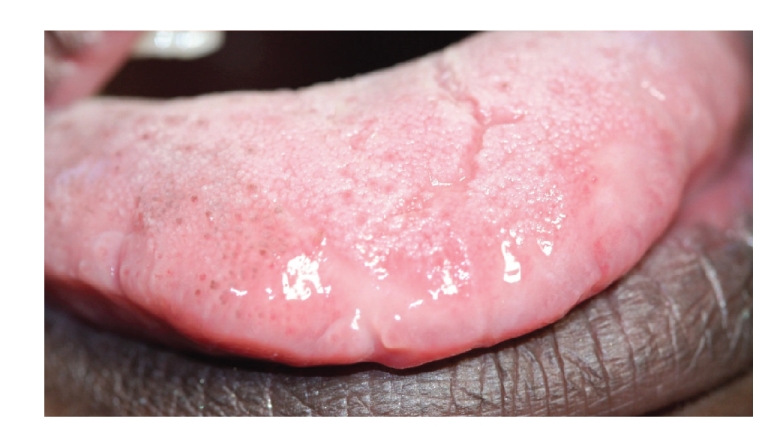
The same region as shown in Figure 2, four weeks after starting antitubercular treatment.

Four weeks after initiation of the anti-TB treatment the oral lesions were strikingly improved (Figures 5 and 6), and 8 weeks later the previously affected lip and tongue looked normal.

## 3. Discussion

M.tb is a slowly-growing, aerobic, nonmotile, noncapsulated acid-fast bacillus. It is a slowly replicating intracellular pathogen in macrophages that elicits a T cell immune response mediated by antigen-specific CD4+ and CD8+ T cells. This immune response may eliminate the M.tb, but more frequently M.tb persists in a latent form, constituting a reservoir of inactive M.tb that under certain circumstances may become active. Only 5%–10% of subjects with latent M.tb infection will develop active TB, but in the remainder the infection will remain inactive and the infected subjects will remain asymptomatic for life. Subjects with immunosuppressing conditions and children with immature immunity are at higher risk of developing active TB than immunocompetent subjects. Infection with HIV is the greatest single risk factor either for the progression of latent infection to active TB or for acquisition of new M.tb infection [1].

The mechanisms that prevent activation of latent M.tb, and those that bring active TB infection under control are poorly characterized [13]. From research done on the pathogenesis of TB in animals it appears that CD4+ T cells play an essential role in controlling active M.tb infection, but only a minor role in preventing re-activation of latent M.tb infection. On the other hand, CD8+ T cells provide immunity against re-activation of latent infection, but make only a limited contribution to the containment of active M.tb infection [13].

In the process of primary M.tb infection, the mycobacteria are phagocytosed by macrophages which activate CD4+ T cells. Subsequently both macrophages and CD4+ T cells secrete a variety of cytokines that in turn recruit and activate lymphocytes and mononuclear phagocytes, which fuse into multi-nucleated giant cells and generate the typical T cell mediated immunoinflammatory granuloma (tubercle) that contains the M.tb microorganisms [1, 13]. Reactive nitric oxide metabolites produced by activated macrophages play an important role in the intracellular neutralization of the bacteria [1, 3].

CD8+ cytotoxic T effector cells recognize M.tb antigens on infected macrophages in the context of MHC class 1 molecules and induce either killing of the intracellular pathogens or lysis of the infected cells by means of granzymes, granulysin or perforin. Furthermore a Th-1 cytokine profile is important for building up a protective immunoinflammatory response to M.tb infection [1].

HIV-seropositive subjects are at greater risk of activation of latent M.tb infection, of acquiring new M.tb infection, including drug-resistant M.tb, and of rapid progression of active TB disease, than are HIV-seronegative subjects [2, 4]. There is a synergistic relationship between tuberculosis and HIV infection: each accelerates the progression of the other; and HIV-seropositive subjects with TB have a shorter life than TB-free HIV-seropositive subjects with comparable CD4+ T cell counts [2].

The pathogenesis of HIV-M.tb co-infection is complex and multifactorial. The profound HIV-associated cellular immune suppression has several identifiable characteristics: a progressive decrease of CD4+ T cells, functional impairment of surviving CD4+ T cells, functional impairment of macrophages and polymorphonuclear neutrophils, dysregulation of the cytokine network and CD8+ T cell exhaustion. These factors in concert promote activation of latent M.tb infection and acquisition of new M.tb from exogenous sources. In turn, M.tb-specific chronic activation of the cellular arm of the immune response adds to the existing HIV-associated CD4+ T-cell and CD8+ T-cell exhaustion. Moreover, M.tb promotes HIV replication by upregulating CXCR4 surface receptors on alveolar macrophages thus permitting the more virulent X4 strains of HIV to enter and replicate in these cells. This leads to further immune exhaustion and impairment, resulting in the perpetuation of a pernicious cycle of HIV-M.tb co-infection [2, 14].

If highly active antiretroviral therapy (HAART) is introduced early in the course of HIV disease and if concurrent TB is treated expeditiously, then this pernicious cycle will be arrested. It is important to note that the outcome of treatment of TB in either HIV-seropositive subjects or HIV-seronegative subjects is similar, but recurrence of, and mortality rates from TB are greater in HIV-seropositive subjects [2].

TB may occur at any stage of HIV disease and, as in the case presented here, may be the first indicator of HIV infection. TB may also sometimes present as an immune reconstitution inflammatory syndrome shortly after HAART has brought about a decrease in the HIV load with consequential significant elevation of the CD4+ T cell count [2].

HIV-seropositive subjects and particularly those HIV-seropositive subjects with low CD4+ T cell counts more frequently have extrapulmonary TB than do HIV-seronegative subjects or HIV-seropositive subjects with high CD4+ T cell counts [2]. Primary extrapulmonary TB is much less common than secondary extrapulmonary TB, and primary oral TB as the sole manifestation of TB in an HIV-seropositive subject, as in our patient, is rare.

The diagnosis of TB in HIV-seropositive subjects is not always straightforward as the clinical signs and symptoms of TB in these subjects are not as well defined as in HIV-seronegative subjects. A comprehensive discussion of the signs and symptoms of HIV-M.tb co-infection is beyond the scope of this paper, but it is important to note that HIV-M.tb co-infected subjects are frequently negative to tuberculin skin testing, acid fast bacilli are very scant in their sputum although sputum culture invariably confirms pulmonary TB, and pulmonary TB granulomas are not always present [2].

The diagnosis of primary oral TB in our patient was made by biopsy since the clinical features of the oral lesions were nonspecific. On microscopical examination, typical tuberculous granulomas were not evident. The presence of epithelioid cells prompted the Ziehl-Neelsen stain which revealed the acid-fast bacilli. 

Failure to express well-defined granulomas with giant cells is the result of immune suppression due to HIV co-infection. Following anti-TB treatment the lesions resolved. Unfortunately the patient was denied antiretroviral treatment because in government hospitals in South Africa this is allowed only to patients whose CD4+ T cell counts are lower than 200 cells/mm^3^, but our patient had a CD4+ T cell count of 429 cells/mm^3^. Unfortunately our patient could not afford the medication privately. 

It would have been academically instructive to have performed microbiological culture and/or PCR investigation to determine the specific type of mycobacterium in our case. However, in the resource-poor area in which our hospital is situated in South Africa these tests are not done routinely. 

However, since all milk products in the urban area where the patient lives are pasteurized, and since mycobacterial species other than M.tb are uncommonly encountered in this geographic region, it is reasonable to assume that our patient was infected with M.tb.
